# Supplementary material for: Antiproliferative Benzoindazolequinones as Potential Cyclooxygenase-2 Inhibitors
Source: Molecules. 2019 Jun 18;24(12):2261. doi: 10.3390/molecules24122261 (PMC6630654; doi:10.3390/molecules24122261)
Supplement: Supplementary file 1 [file molecules-24-02261-s001.zip › supple/Figure S1.docx]

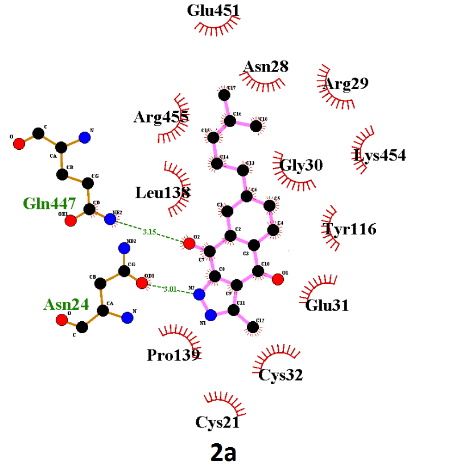


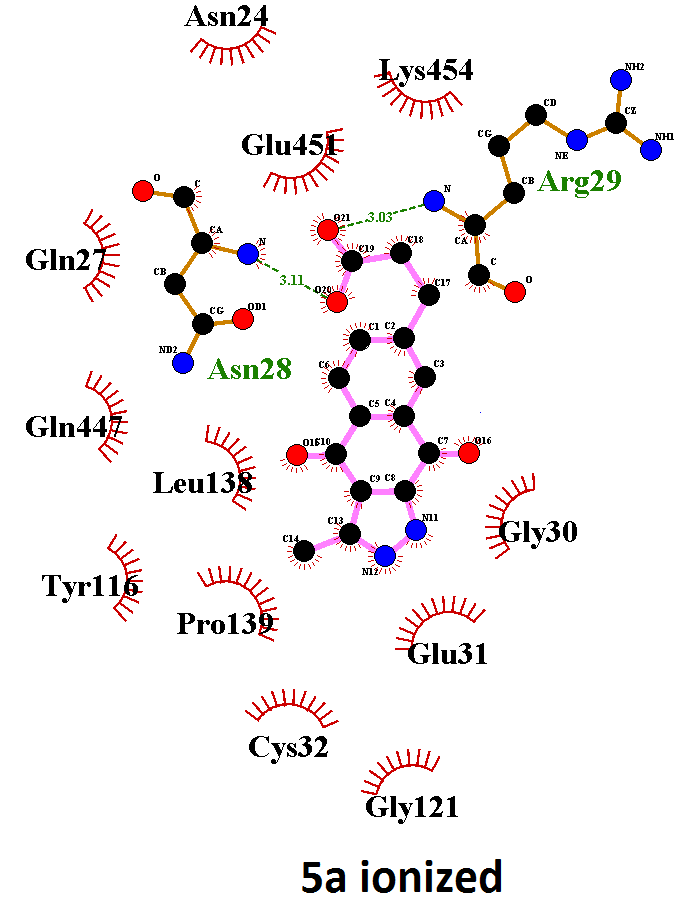


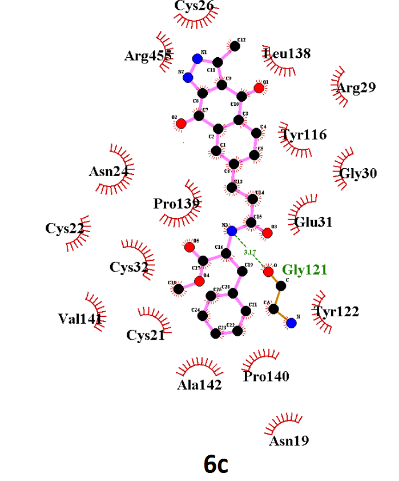


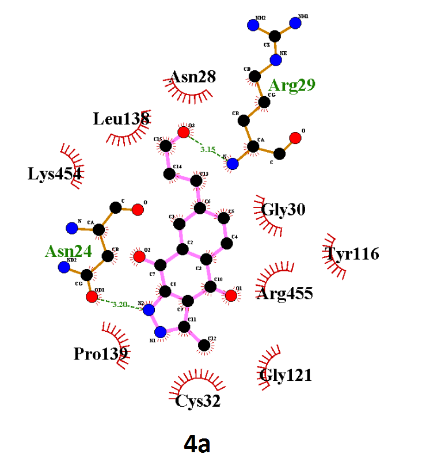


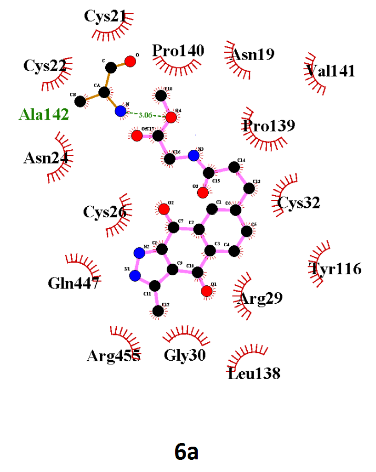


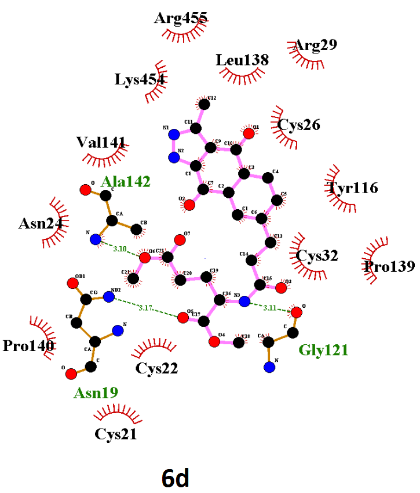


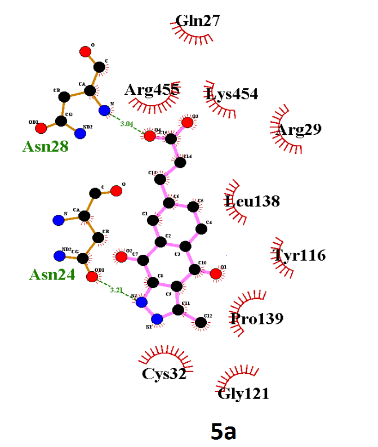


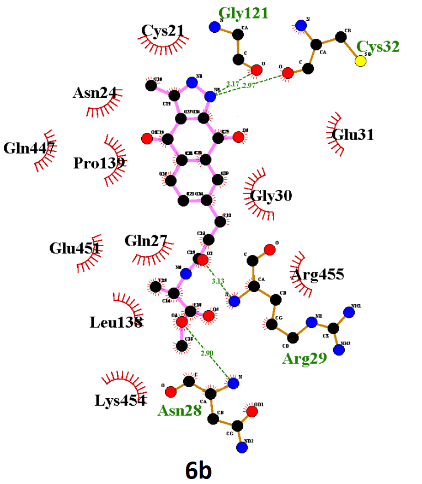


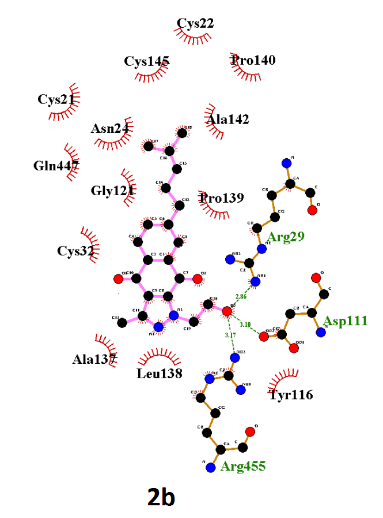


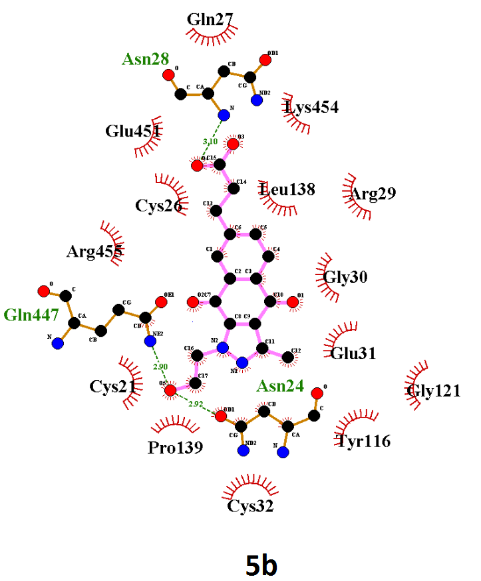


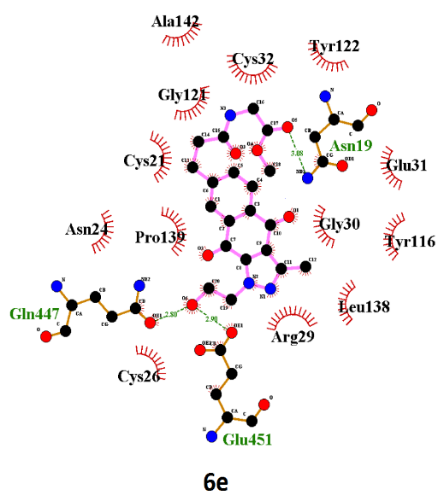


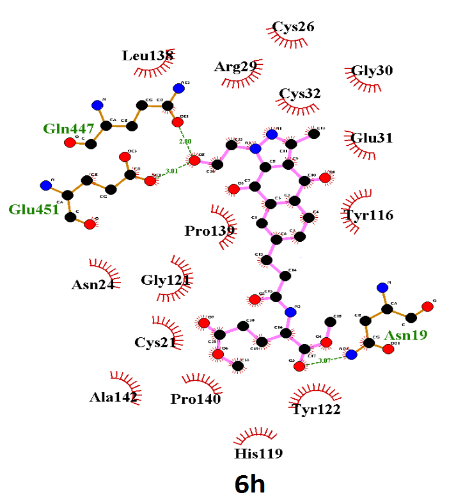


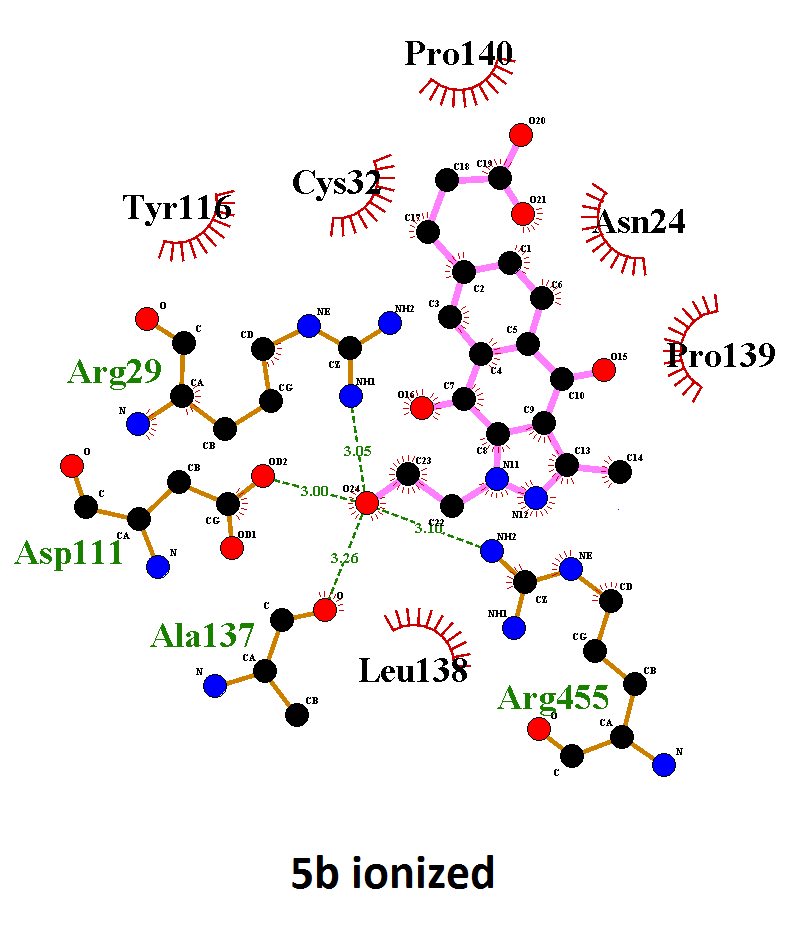


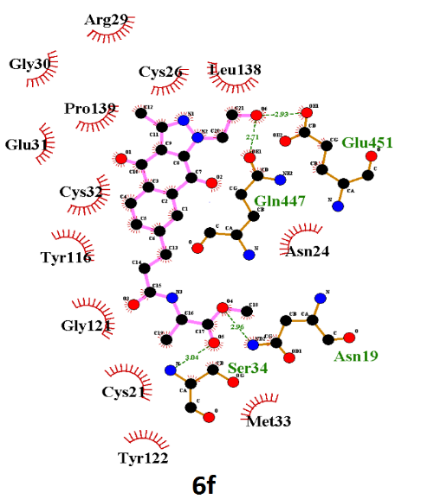


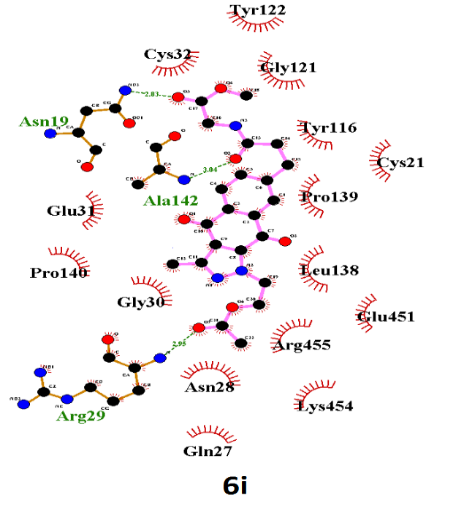


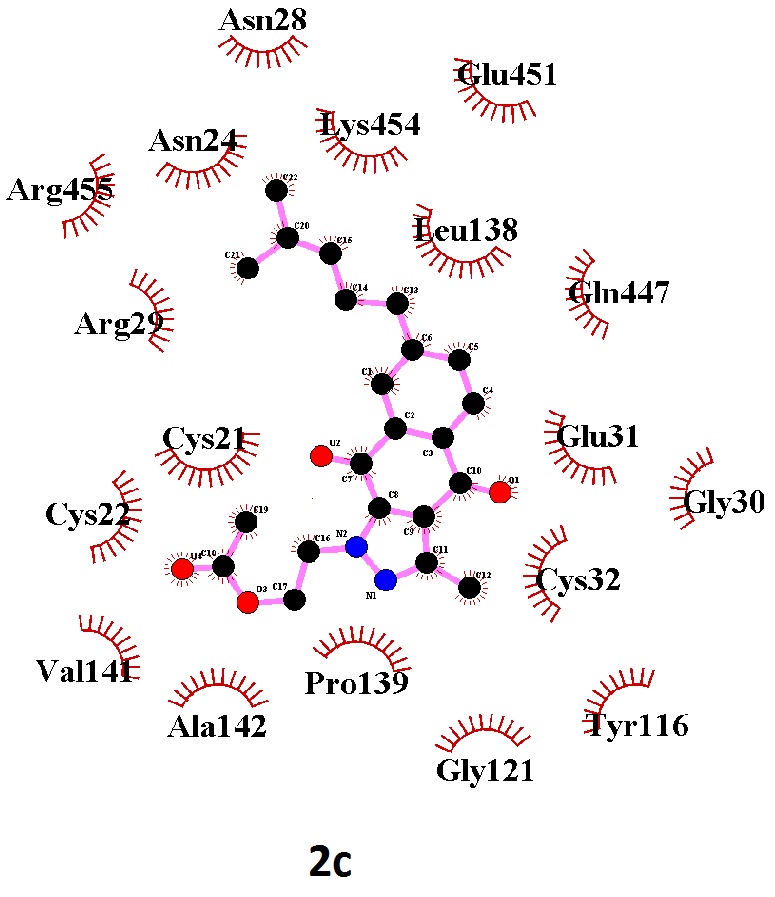


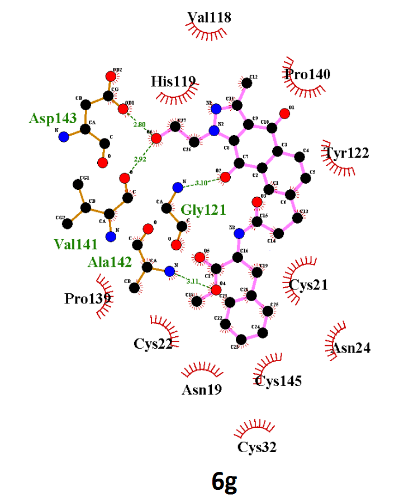


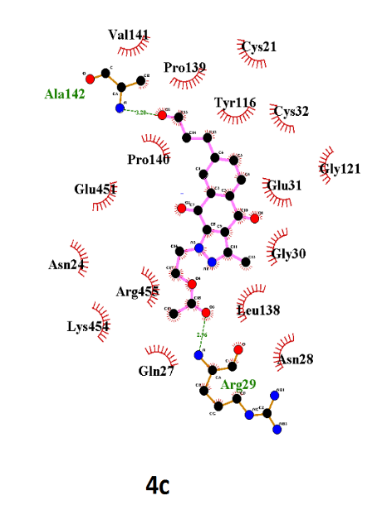


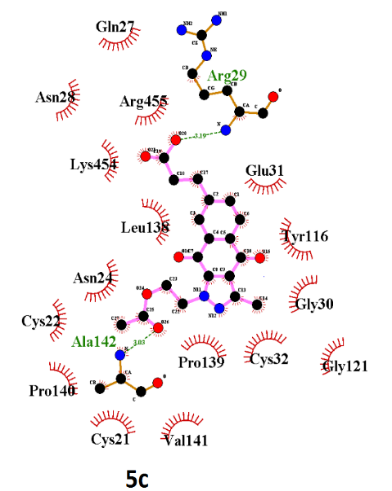


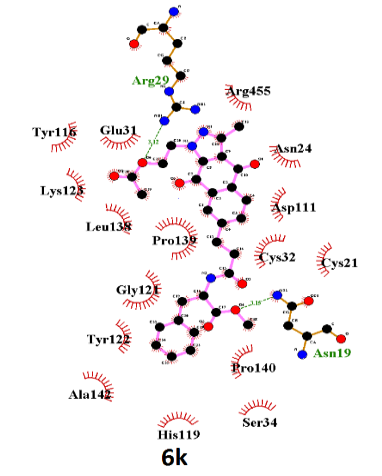


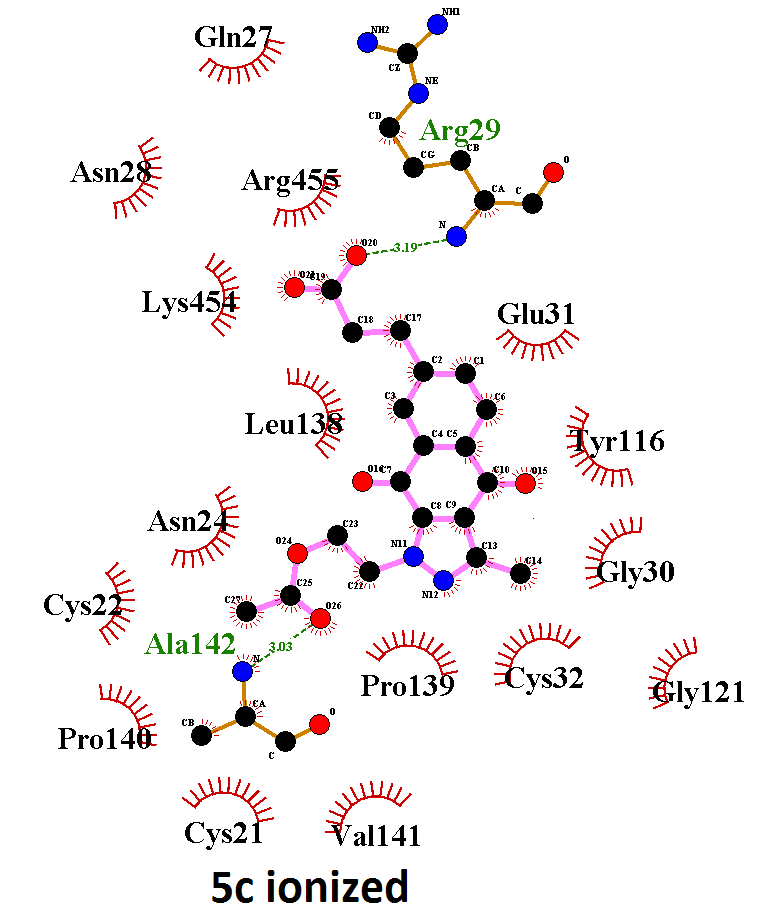


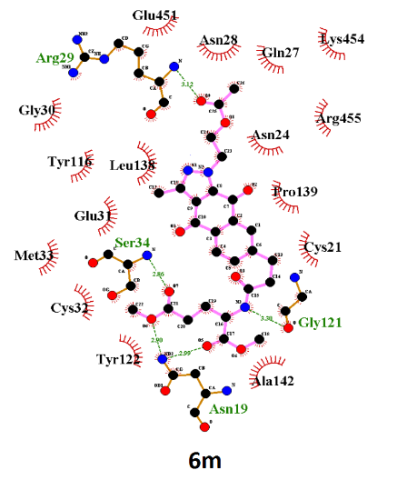


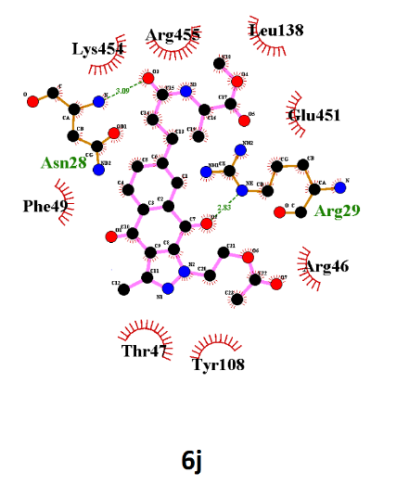


Figure S1: 2D-maps of hydrogen-bond interaction patterns and hydrophobic contacts between some BIZQs and the main-chain or side-chain elements of COX-2 protein.
