# Supplementary material for: Antiproliferative Benzoindazolequinones as Potential Cyclooxygenase-2 Inhibitors
Source: Molecules. 2019 Jun 18;24(12):2261. doi: 10.3390/molecules24122261 (PMC6630654; doi:10.3390/molecules24122261)
Supplement: Supplementary file 1 [file molecules-24-02261-s001.zip › supple/Table S1.docx]

| **Table ST1. Enzyme Inhibition Scores as Predicted by Molinspiration** ^a^ **Algorithm** | | | | | |  |
| --- | --- | --- | --- | --- | --- | --- |
| ***Subseries a* (2a - 5c)** | | |  |  | |  |
|  | | *in vitro* pIC_50_ | *in vitro* pIC_50_ |  |  |  |
| **Comp.** | | **KATO** | **MCF7** | **Enzyme inhb.** | **Protease inhb.** | **Kinase inhb.** |
| **2a** | | **4.22** | **4.20** | **0.52** | -0.01 | **0.22** |
| **2b** | | **4.59** | **4.56** | **0.44** | -0.07 | **0.12** |
| **2c** | | **4.48** | **4.53** | **0.38** | -0.08 | -0.02 |
|  | |  |  |  |  |  |
| **3a** | | 3.50 | 3.36 | **0.63** | **0.26** | **0.12** |
| **3b** | | 3.40 | 3.38 | **0.54** | **0.16** | 0.02 |
| **3c** | | 3.49 | 3.41 | **0.48** | **0.12** | -0.10 |
|  | |  |  |  |  |  |
| **4a** | | **4.00** | 3.91 | **0.52** | **0.10** | **0.20** |
| **4b** | | **4.20** | **4.36** | **0.44** | 0.07 | **0.14** |
| **4c** | | **4.22** | **4.48** | **0.37** | 0.08 | -0.02 |
|  | |  |  |  |  |  |
| **5a** | | 3.49 | 3.43 | **0.52** | 0.03 | **0.19** |
| **5b** | | 3.47 | 3.48 | **0.40** | -0.05 | **0.10** |
| **5c** | | 3.79 | 3.61 | **0.36** | -0.04 | 0.03 |
|  | |  |  |  |  |  |
| ***Subseries b* (6a - 6m)** | | |  |  | |  |
|  | | *in vitro* pIC_50_ | *in vitro* pIC_50_ |  |  |  |
| **Comp.** | | **KATO** | **MCF7** | **Enzyme inhb.** | **Protease inhb.** | **Kinase inhb.** |
| **6a** | | 3.64 | 3.38 | **0.22** | 0.09 | -0.05 |
| **6e** | | 3.51 | 3.54 | **0.19** | -0.01 | -0.12 |
| **6i** | | 3.68 | 3.59 | **0.15** | -0.05 | -0.19 |
|  | |  |  |  |  |  |
| **6b** | | 3.94 | **4.03** | **0.24** | **0.15** | -0.03 |
| **6f** | | **4.36** | **4.20** | **0.21** | 0.05 | -0,10 |
| **6j** | | **4.27** | **4.28** | **0.17** | 0.01 | -0.16 |
|  | |  |  |  |  |  |
| **6c** | | 3.90 | 3.81 | **0.25** | **0.18** | 0.00 |
| **6g** | | **4.42** | **4.41** | **0.22** | 0.09 | -0.07 |
| **6k** | | **4.46** | **4.45** | **0.15** | 0.04 | -0.17 |
|  | |  |  |  |  |  |
| **6d** | | 3.95 | 3.84 | **0.30** | **0.17** | -0.04 |
| **6h** | | **4.28** | 4.06 | **0.26** | 0.08 | -0.11 |
| **6m** | | 3.96 | 4.00 | **0.21** | 0.04 | -0.17 |
| 1. Molinspiration *version 2018.10* 2. IC_50_  expressed in mol/L 3. Bold numbers correspond to the best values of *in vitro* and *in silico* activity | | | | | | |
|  | | | | | | |
