# Supplementary material for: Antiproliferative Benzoindazolequinones as Potential Cyclooxygenase-2 Inhibitors
Source: Molecules. 2019 Jun 18;24(12):2261. doi: 10.3390/molecules24122261 (PMC6630654; doi:10.3390/molecules24122261)
Supplement: Supplementary file 1 [file molecules-24-02261-s001.zip › supple/Table S2.docx]

| T . Table ST2. Pharmacokinetic data predicted by QikProp^a^ for BIZQ derivatives of Series I, II and III | | | | | | | | |
| --- | --- | --- | --- | --- | --- | --- | --- | --- |
| Compd. | Percent Human Oral Absorption^b^ | QPPCaco^c^ | QPlogBB^d^ | QPlogHERG^e^ | QPlogKhsa^f^ | QPlogS^g^ | PSA^h^ | N of  violations (<3) |
| Series Ia |  |  |  |  |  |  |  |  |
| 2a | 88.36 | 373.21 | -1.06 | -4.83 | 0.27 | -4.89 | 86.87 | 0 |
| 3a | 83.37 | 373.49 | -0.99 | -4.42 | -0.11 | -3.66 | 99.95 | 0 |
| 4a | 61.59 | 64.92 | -1.69 | -4.32 | -0.45 | -2.71 | 125.35 | 0 |
| 5a | 48.08 | **8.73** | -1.97 | -2.57 | -0.52 | -3.01 | 138.53 | 1 |
| Series Ib |  |  |  |  |  |  |  |  |
| 6a | 50.64 | **16.01** | -2.64 | -4.12 | -0.55 | -3.49 | 163.30 | 1 |
| 6b | 60.61 | 39.17 | -2.27 | -4.11 | -0.43 | -4.07 | 157.98 | 0 |
| 6c | 71.81 | 47.56 | -2.46 | **-5.34** | 0.05 | -5.68 | 155.51 | 1 |
| 6d | 52.63 | **12.75** | **-3.33** | -4.70 | -0.42 | -4.96 | 193.07 | 2 |
|  |  |  |  |  |  |  |  |  |
| Series IIa |  |  |  |  |  |  |  |  |
| 2b | 90.65 | 426.90 | -1.27 | **-5.21** | 0.17 | -4.82 | 93.79 | 1 |
| 3b | 85.55 | 426.92 | -1.20 | -4.85 | -0.23 | -3.78 | 106.76 | 0 |
| 4b | 63.75 | 74.18 | -1.95 | -4.74 | -0.57 | -2.83 | 132.04 | 0 |
| 5b | 50.38 | **9.99** | -2.25 | -2.99 | -0.62 | -3.17 | 145.33 | 1 |
| Series IIb |  |  |  |  |  |  |  |  |
| 6e | 53.04 | **18.83** | -2.87 | -4.40 | -0.67 | -3.59 | 170.22 | 1 |
| 6f | 62.75 | 44.77 | -2.51 | -4.42 | -0.55 | -4.18 | 164.77 | 1 |
| 6g | 73.96 | 54.28 | -2.70 | **-5.62** | -0.06 | -5.79 | 162.31 | 1 |
| 6h | 41.71 | **14.47** | **-3.60** | -4.96 | -0.55 | -5.05 | 199.75 | 2 |
| Series IIIa |  |  |  |  |  |  |  |  |
| 2c | 92.48 | 337.24 | -1.54 | **-5.83** | 0.27 | -5.81 | 109.53 | 1 |
| 3c | 86.50 | 337.32 | -1.47 | **-5.52** | -0.27 | -4.43 | 122.53 | 1 |
| 4c | 64.36 | 58.65 | -2.31 | **-5.45** | -0.67 | -3.35 | 147.79 | 1 |
| 5c | 52.86 | **7.88** | -2.64 | -3.68 | -0.40 | -4.42 | 161.09 | 1 |
| Series IIIb |  |  |  |  |  |  |  |  |
| 6i | 54.83 | **14.45** | **-3.29** | -4.94 | -0.52 | -4.68 | 185.96 | 2 |
| 6j | 64.88 | 35.39 | -2.87 | -4.93 | -0.40 | -5.28 | 180.54 | 0 |
| 6k | 63.44 | 42.95 | **-3.05** | **-6.08** | 0.14 | **-7.01** | 178.07 | 3 |
| 6m | 30.83 | **11.46** | **-4.01** | **-5.39** | -0.41 | -6.12 | 215.50 | 3 |
| ^a^ QikProp (QP) version 4.3; ^b^ > 80% -high and <25% - poor ; ^c^ QPPCaco: Predicted apparent Caco-2 cell permeability, <25 nm/sec - poor and >500 nm/sec - great; ^d^ QPlogBB: Predicted brain/blood partition coefficient, appropriate values between -3.0 and 1.2; ^e^ QPlogHERG: Predicted IC50 value for blockage of HERG K+ channels, above −5; ^f^ QPlogKhsa: Prediction of binding to human serum albumin, appropriate values between -1.5 and 1.5; ^g^ QPlogS: Predicted aqueous solubility, appropriate values between -6.5 and 5 mol/L; ^h^ PSA: polar surface area, appropriate values between 70–200 Å^2^ . The intervals of appropriate values are defined by QP. Bold numbers correspond to violations of Jorgensen Rule-of-Three | | | | | | | | |
