# Supplementary material for: Antiproliferative Benzoindazolequinones as Potential Cyclooxygenase-2 Inhibitors
Source: Molecules. 2019 Jun 18;24(12):2261. doi: 10.3390/molecules24122261 (PMC6630654; doi:10.3390/molecules24122261)
Supplement: Supplementary file 1 [file molecules-24-02261-s001.zip › supple/Table S3.docx]

| Table ST3. Evaluation parameters of Lipinski’s rule predicted by QikProp^a^ of five and its extensions for BIZQs derivatives from Series I, II and III | | | | | | |
| --- | --- | --- | --- | --- | --- | --- |
| Compd. | mol_MW (<500amu) | donorHB (<5) | accptHB (<10) | QPlogPo/w (<5) | #rotor (0−15) | N of violations (<5) |
| Series I |  |  |  |  |  |  |
| 2a | 294.35 | 1.00 | 5.00 | 2.62 | 3 | 0 |
| 3a | 310.35 | 1.00 | 7.00 | 1.77 | 3 | 0 |
| 4a | 268.27 | 1.00 | 7.00 | 0.37 | 3 | 0 |
| 5a | 284.27 | 2.00 | 7.00 | 0.73 | 3 | 0 |
| 6a | 355.34 | 1.25 | 8.75 | 0.36 | 5 | 0 |
| 6b | 369.37 | 1.25 | 8.75 | 0.88 | 5 | 0 |
| 6c | 445.47 | 1.25 | 8.75 | 2.53 | 7 | 0 |
| 6d | 441.44 | 1.25 | 10.75 | 1.01 | 8 | 0 |
| Series II |  |  |  |  |  |  |
| 2b | 338.40 | 1.00 | 6.70 | 2.84 | 6 | 0 |
| 3b | 354.40 | 1.00 | 8.70 | 1.96 | 6 | 0 |
| 4b | 312.32 | 1.00 | 8.70 | 0.57 | 6 | 0 |
| 5b | 328.32 | 2.00 | 8.70 | 0.94 | 6 | 0 |
| 6f | 413.42 | 1.25 | 10.45 | 1.07 | 8 | 0 |
| 6e | 399.40 | 1.25 | 10.45 | 0.56 | 8 | 0 |
| 6g | 489.52 | 1.25 | 10.45 | 2.72 | 10 | 0 |
| 6h | 485.49 | 1.25 | **12.45** | 1.18 | 11 | 1 |
| Series III |  |  |  |  |  |  |
| 2c | 380.44 | 0.00 | 7.00 | 3.46 | 6 | 0 |
| 3c | 396.44 | 0.00 | 9.00 | 2.44 | 6 | 0 |
| 4c | 354.36 | 0.00 | 9.00 | 0.98 | 6 | 0 |
| 5c | 370.36 | 1.00 | 9.00 | 1.68 | 6 | 0 |
| 6i | 441.44 | 0.25 | 10.75 | 1.21 | 8 | 0 |
| 6j | 455.46 | 0.25 | 10.75 | 1.74 | 8 | 0 |
| 6k | **531.56** | 0.25 | 10.75 | 3.45 | 10 | 1 |
| 6m | **527.53** | 0.25 | **12.75** | 1.85 | 11 | 2 |
| ^a^ QikProp (QP) version 4.3; mol_MW: Molecular weight; donorHB: Number of hydrogen-bond donors (OH, NH, SH); accptHB: Number of hydrogen-bond acceptors (heteroatom O; N; S; F or pairs of transferable electrons); QPlogPo/w: Predicted *n-*octanol/water partition coefficient; #rotor: Number of non-trivial (not CX3), non-hindered (not alkene, amide, small ring) rotatable bonds. Bold numbers correspond to Lipinski Rule-of Five violations | | | | | | |
